# Identification and characterization of nucleotide metabolism and neuroendocrine regulation-associated modification patterns in stomach adenocarcinoma with auxiliary prognostic assessment and immunotherapy response prediction

**DOI:** 10.3389/fendo.2022.1076521

**Published:** 2023-01-16

**Authors:** Yong Zhang, Lingfeng Zeng, Dexin Lin, Guijian Chang, Yueyue Zeng, Yueming Xia

**Affiliations:** ^1^ Department of General Surgery, Ningde Municipal Hospital of Ningde Normal University, Ningde, China; ^2^ Carol & Richard Yu Peritoneal Dialysis Research Centre, Department of Medicine & Therapeutics, Prince of Wales Hospital, Shatin, Hong Kong SAR, China; ^3^ Li Ka Shing Institute of Health Sciences (LiHS), Faculty of Medicine, The Chinese University of Hong Kong, Shatin, Hong Kong SAR, China

**Keywords:** stomach adenocarcinoma, nucleotide metabolism, neuroendocrine regulation, molecular features, immunotherapy, tumor microenvironment

## Abstract

**Background:**

The significance of nucleotide metabolism and neuroendocrine in cellular immune response and cancer is becoming more well-established. However, the mechanisms underlying nucleotide metabolism and neuroendocrine involvement in stomach adenocarcinoma (STAD) remain unclear.

**Methods:**

First, a pan-cancer overview of nucleotide metabolism and neuroendocrine-related genes (NMNGs) was explored through the integration of expression profiles, prognostic values, mutation information, methylation levels, and pathway-regulation relationships. We next extensively assessed variations in prognosis and tumor microenvironment (TME) features across the various modification patterns, based on an extensive analysis of the NMNG modification patterns of 808 STAD samples based on 46 NMNGs. Utilizing principal component analysis methodologies, the NMNGscore was developed to measure NMNG alteration patterns of individual tumors.

**Results:**

Pan-cancer analysis shows that NMNGs mostly act as risk genes in multiple cancer types, especially in STAD. Based on the NMNGs we detected two different NMNG modification patterns in STAD. Both patterns showed distinct immune cell infiltration features and biological behavior, with NMNGcluster A exhibiting a worse prognosis and a larger amount of immune infiltration. Differentially expressed genes with prognostic relevance were used to classify the STAD samples into three genomic subgroups. Analysis of survival rates revealed that cluster B genes were associated with longer life expectancy than clusters A and C. Individual STAD patients’ NMNG alteration patterns were analyzed by analyzing their NMNGscore signatures. NMNGscore and immune cells showed a statistically significant adverse correlation with each other. Increased longevity, a higher incidence of mutations, and a better response to immunotherapy were associated with patients’ NMNG scores.

**Conclusions:**

Our findings provide a personalized prediction tool for prognosis and immunotherapy sensitivity in patients, as well as a promising knowledge of nucleotide metabolism and neuroendocrine in STAD.

## Introduction

Stomach adenocarcinoma (STAD) is the most common pathological tissue type of gastric cancer (GC) and the third leading cause of cancer-related death (1). Every year, the number of new cases of GC in China accounts for 47% of all GC cases worldwide. More than 60% of patients are either locally advanced or late when they begin therapy, and fewer than 30% will be alive after 5 years (2, 3). Surgery, chemotherapy, and radiation are inadequate treatments for stomach cancer. Immunotherapy based on immune checkpoint blocking (ICB, PD-1/L1, and CTLA-4) has demonstrated incredible therapeutic results in a small subset of patients with durable responses. However, the majority of individuals have little to no therapeutic improvement (4). Therefore, the development of suitable biomarkers is crucial for predicting the prognosis and treatment success of GC patients.

Metabolic reprogramming is regarded as one of the hallmarks of cancer, contributing to the process of cancer incidence, progression, and metastasis. Nucleotide metabolism constitutes the final and most crucial link in the chain of events that contribute to the spread of cancer (5). To achieve uncontrolled cell proliferation, tumor cells use the nucleotide metabolism pathway to synthesize DNA and RNA (6). It has been reported that UHMK1 is able to promote GC progression by reprogramming nucleotide metabolism (7). Besides speeding up tumor formation, recent research has demonstrated that aberrant nucleotide metabolism also dampens the normal immune response in the tumor microenvironment (TME). For example, disrupting the homeostasis of the pools of nucleotides can produce mutations that influence antigen presentation and, ultimately, the immune response to the tumor (8, 9). Targeting nucleotide metabolism also provides new directions, for the development of novel antitumor-specific drugs (10, 11). Furthermore, the onset and advancement of several cancers are significantly influenced by neuroendocrine pathways (12). Cancers of the gastric, liver, pancreas, colorectal, breast, and uterus are all thought to be linked to neuroendocrine regulation disorders such as diabetes, obesity, and depression. For example, catecholamine-induced neuroendocrine phenotypes of GC cells led to depression-accelerated GC invasion and metastasis (13). In addition, chronic stress has been linked to CNS disorders as well as tumor onset and development, according to several studies (14, 15). The development of tumors caused by stress is significantly influenced by immune regulation (16). According to research, long-term stress causes broad immune cell modification in tissues, resulting in immunological suppression or dysregulation (17, 18). In addition, neuroendocrine could be able to control metabolism through hormones and neurotransmitters (19). Meanwhile, the nucleotide metabolism and neuroendocrine-related genes (NMNGs) have not been discovered to predicate clinical outcomes and treatment strategies in patients with STAD according to our best knowledge. Thus, the development of the STAD prognosis signature using NMNGs is promising.

In this study, we first analyzed the genetic variations and expression patterns of NMNGs in numerous cancer types. Next, we used genomic information from 808 STAD cases to characterize TMEs and analyze the patterns of NMNG alteration. By subsequent analysis, the sample was divided into two categories with significantly different prognoses and TMEs, indicating that NMNG modifications had a significant effect on the development of individual TME characteristics. In addition, a set of scoring methods intended to assess the pattern of NMNG alteration in people was developed, taking into account the variety of NMNG modification among individuals.

## Materials and methods

### Data collection and processing

The datasets on STAD, which contain 32 normal and 375 tumor samples, were queried in the Cancer Genome Atlas (TCGA) for the purpose of extracting information on gene expression and clinical annotations. The GSE84437 cohort of the Gene Expression Omnibus (GEO) database had 433 tumor samples. Batch normalization was implemented using the R package “sva” (20). We also collected data from the TCGA platform on single nucleotide variation (SNV), transcriptome profiles, copy number variation (CNV), methylation, and clinical characteristics of pan-cancer transcriptomes. In addition, 4594 nucleotide metabolism-related genes and 271 neuroendocrine-related genes (relevance score>2.5) were obtained from the GeneCard Database (https://www.genecards.org/). After taking the intersection of the two groups of genes, 196 NMNGs were obtained and displayed by Venn diagram. The TCGA and GEO cohorts were screened for NMNGs with prognostic values using univariate cox regression for subsequent analysis. The prognostic significance of them was also validated using Kaplan-Meier (KM) analysis based on 808 STAD samples.

### Pan-cancer analysis

There has been minimal investigation on the relationship between nucleotide metabolism, neuroendocrine, and cancers yet. Consequently, the distinctions between NMNGs in diverse cancers are inadequately documented. SNV, CNV, methylation, and mRNA expression data were analyzed and visually displayed as heatmaps to offer an overview of NMNGs across all cancer types. A univariate Cox regression analysis between mRNA expression and cumulative survival was carried out to further assess the involvement of NMNGs in the prognosis of various cancers. The potential impact of NMNG on the traditional oncogenic pathway of pan-cancer was then investigated using the single sample gene set enrichment analysis (ssGSEA) (21). All of these analyses were carried out using R as in earlier published studies (22, 23).

### Cluster analysis of NMNG

Unsupervised clustering analysis was done to identify distinctive NMNG modification patterns and divided STAD samples into different categories based on the expression of prognostic NMNGs. A consensus clustering procedure was conducted to determine the number and stability of each individual cluster, and the “Consensus Cluster Plus” software package provided additional support (24). The clustering effect indicated that the clustering stability was improved with k=2.

### Gene set variation analysis (GSVA) and ssGSEA

The “GSVA” R package was used to conduct GSVA enrichment analysis so as to investigate differences in biological processes across various NMNG modification patterns. We used ssGSEA to evaluate a large number of immune cells (25), such as activated dendritic cells, activated CD4 T cells, activated CD8 T cells, and activated B cells, that have infiltrated the TME of STAD. According to the results of the ssGSEA analysis, the relative abundance of each TME-invading cell for every sample was represented by the enrichment scores.

### Analysis of genes with differential expression (DEGs) to identify NMNG modification patterns

We filtered out DEGs using the R package “limma” in accordance with two distinct NMNG modification patterns that we have identified. An adjusted P-value < 0.001 was used for the rank criterion. Additionally, we fundamentally annotated DEGs using the R package “clusterProfiler”. Similar to this, the univariate Cox model chose the survival-related DEGs of the two distinct NMNG modification patterns, and cluster analysis based on the DEGs with prognostic significance was carried out with the aid of the R package “Consensus Cluster Plus.” Furthermore, differences (variations) in NMNG expression and patient survival between clusters were characterized.

### Construction of NMNG signature

On the basis of the results, we subsequently created a scoring system for the NMNG. According to relevant research (26), we conducted PCA of the principal components and chose principal components 1 and 2 as feature scores. Then, the NMNG score was computed using the following equation: score = (PCli+PC2i), where ‘i’ stands for the genes related to the NMNG phenotype.

### Genomic and clinical information of immune checkpoint genes (ICGs)

We used the Wilcoxon test to compare the differential expression of immunological checkpoints such as PDCD1, PDCD1LG2, CD40, CD80, and CD276 in groups with low and high NMNG scores. From the Cancer Immunome Atlas Database, we concurrently gathered the Immune Checkpoint Inhibitor (ICI) Immunophenoscore (IPS) file. The immunotherapeutic relevance of the NMNG gene signature was evaluated using IPS, a reliable tool for assessing tumor immunogenicity.

### Statistical analysis

For all statistical analyses, R was utilized. The one-way ANOVA and Kruskal-Wallis test were used to compare the outcomes across multiple groups. The best cutoff score was divided into groups with low- and high-NMNG scores using the “surv-cutpoint” function. The prognostic survival curve was produced using the KM approach. We evaluated the mutation status of people with low- and high-NMNGscore subtypes using the waterfall function in the maftools package (25). For all analyses, the significance of the correlation is determined by the criterion of p < 0.05.

## Results

### Data procession

**Figure 1:**
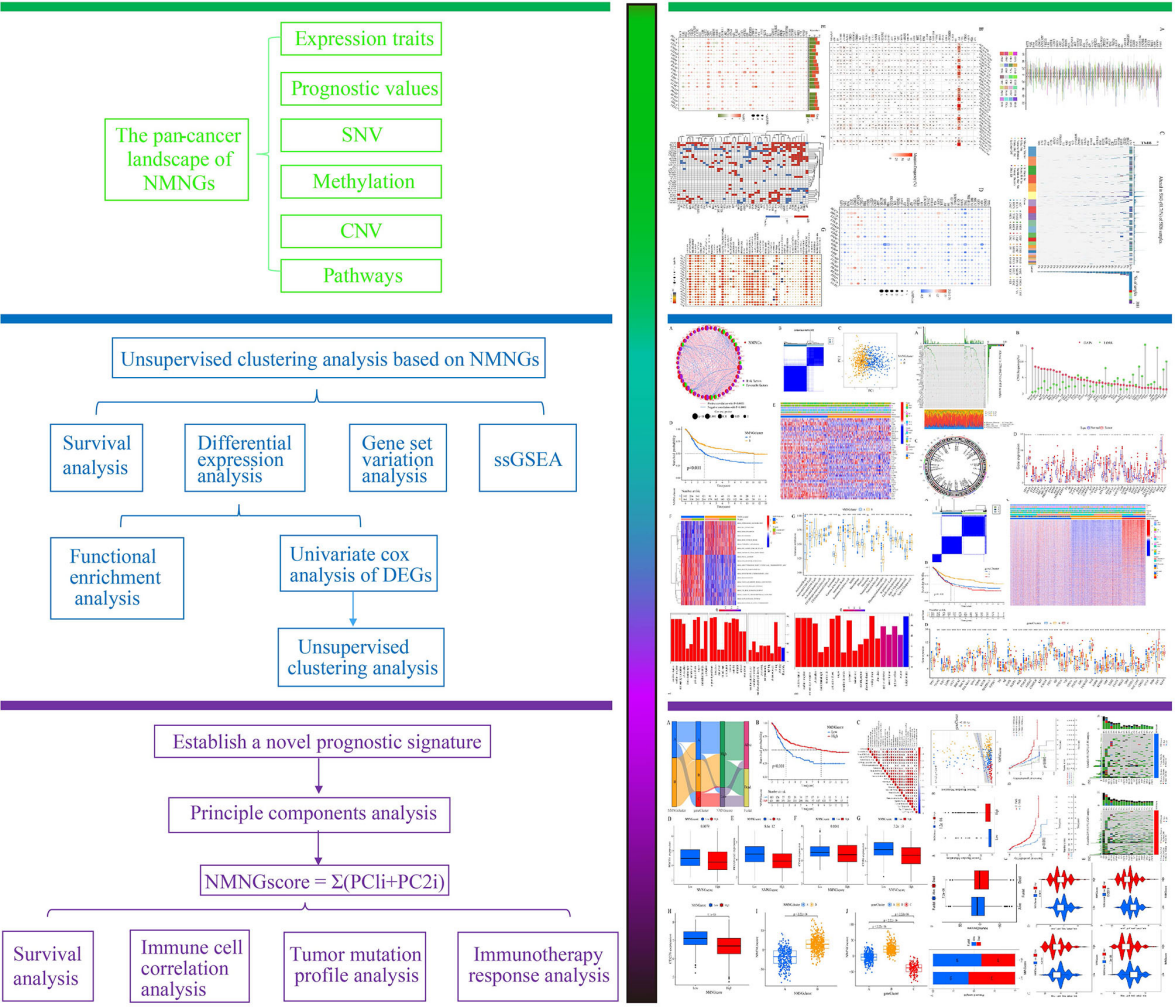
The investigation’s flow chart.

**Figure 1** displays a flowchart outlining the steps in the research process. First, we did the intersection of genes related to neuroendocrine function and genes related to nucleotide metabolism to obtain 196 NMNGs (**Supplementary Figure 1**). The 808 STAD samples were then subjected to a univariate cox regression analysis to yield 46 NMNGs that were prognostically significant for this study (**Supplementary Table S1**). The prognostic significance of 46 NMNGs was validated by KM analysis based on 808 STAD samples (**Supplementary Figure 2**).

### Pan-cancer analysis of 46 NMNGs

**Figure 2:**
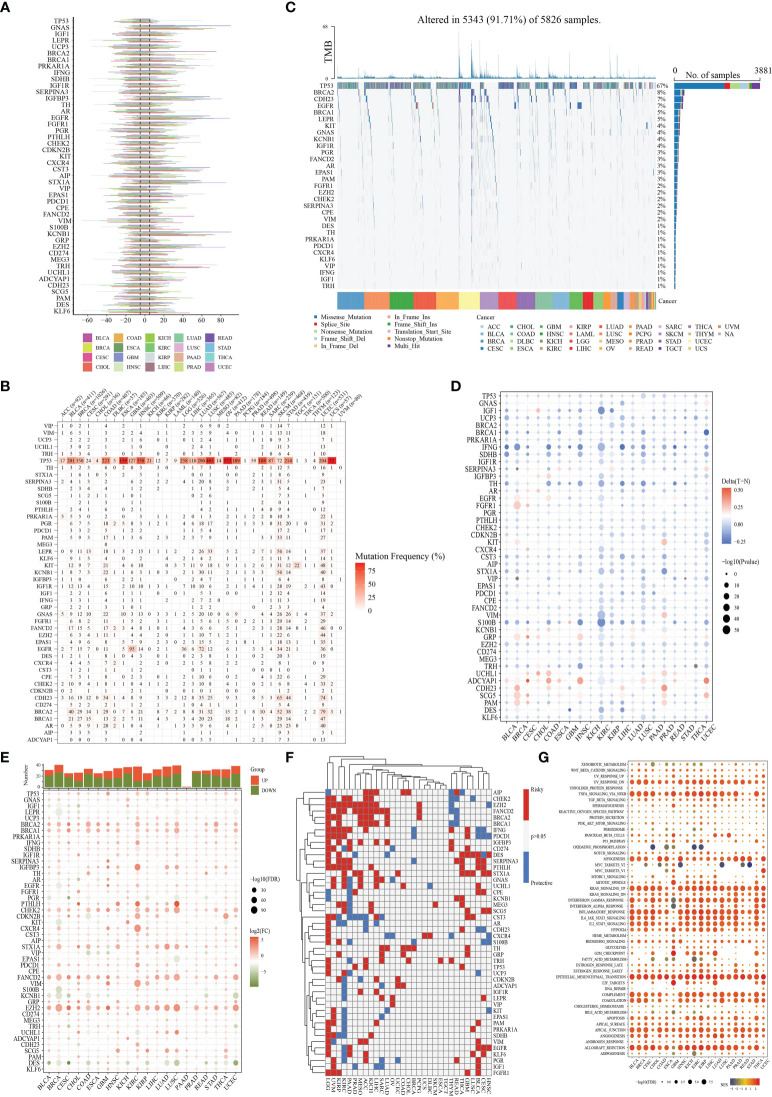
Panoramic view of nucleotide metabolism-related genes (NMNGs) in pan-cancer. **(A)** Histogram shows the frequency of copy number variation (CNV) for each NMNG in each cancer type. **(B)** Mutation frequency of NMNGs. The numbers show how many samples of a certain tumor have the matching mutant gene. “0” denotes the absence of any mutation in the gene’s coding region, while no number denotes the absence of any mutation in the gene at all. **(C)** Single-nucleotide variation (SNV) oncoplot. An oncoplot displaying the distribution of NMNG mutations and a list of SNV kinds. **(D)** Heatmap displays the differential methylation of NMNGs in cancers; hypermethylated and hypomethylated genes are denoted with red and blue, correspondingly (Wilcoxon rank-sum test). **(E)** Histogram (upper panel) and heatmap demonstrate the number of significant DEGs and the fold change and FDR of NMNGs, respectively, in each cancer. Substantially upregulated and downregulated genes are denoted with red and green, correspondingly. **(F)** NMNGs’ survival profiles across cancers. **(G)** Enrichment analysis for cancer pathway signaling between tumor samples with high- and low-NMNGs scores.

Data on CNV, SNV, methylation, mRNA expression profiles, and survival information for 46 NMNGs in various malignancies were provided by TCGA for the pan-cancer study. The percentage of CNV was examined in order to investigate the genetic aberrations of NMNGs in cancer. Different CNV profiles of NMNGs were detected in varied cancer types (**Figure 2A**). For instance, in nearly all tumors, GNAS, IGFBP3, EGFR, STX1A, and KCNB1 were more likely to experience copy number gain than copy number loss, whereas PGR and CDH23 displayed the opposite profile. Moreover, we examined SNV data related to NMNGs to determine the frequency and types of variants in each cancer subtype. **Figure 2B** demonstrates the remarkable SNV of NMNGs in SKCM, STAD, UCEC, LUSC, and LUAD. In addition, 91.71% of the NMNGs had SNV frequency (5343 of 5826 tumors). Missense mutations were discovered to be the primary SNP type as per the variant-type analysis. According to SNV percentage analysis, the top 4 mutated genes were TP53, BRCA2, CDH23, and EGFR, with respective mutation percentages of 67%, 8%, 7%, and 7%. (**Figure 2C**). In addition to CNV, promoter methylation can control gene expression, and abnormal promoter DNA methylation is linked to cancer (27). We noted that most NMNG consistently exhibited hypomethylation in 20 cancer types, with the exception of CDH23, ADCYAP1, and UCHL1 (**Figure 2D**).

For every cancer type, differential expression analysis was done to look into changes in the gene expression patterns of NMNGs in addition to genetic changes between the tumor and nearby normal tissues. Most of the gene expression levels in cancer tissues were different from those in healthy tissues, we discovered, except in pancreatic cancer tissues. In cancer tissue, most NMNGs are expressed at high levels (**Figure 2E**). Univariate Cox regression of mRNA expression and OS was then used to identify risky NMNGs with HR > 1 and pValue < 0.05 as well as protective NMNGs with HR < 1 and pValue < 0.05, as shown in **Figure 2F**. We found that most genes are risk factors for multiple cancer types. Since it is currently unknown how nucleotide metabolism and neuroendocrine regulates pathways connected to cancer, it is imperative to examine any possible connections between these pathways and NMNGs. This will lay the basis for future investigation into how NMNGs control pan-cancer-related pathway regulation. According to our findings, NMNGs in pan-cancer were significantly correlated with a number of signaling pathways, including TNFA signaling *via* NFKB, KRAS signaling, interferon-gamma response, inflammatory response, and epithelial-mesenchymal transition (EMT) (**Figure 2G**).

### Landscape of the genetic variation of NMNG in STAD

**Figure 3:**
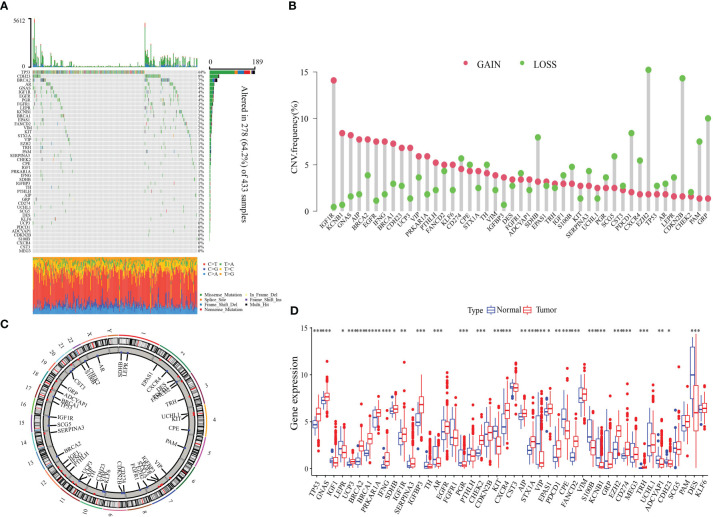
Landscape of genetic and expression variation of NMNGs in STAD. **(A)** 278 of 433 samples have genetic alterations of 46 NMNGs. **(B)** CNV mutation frequency of NMNGs. **(C)** Position of CNV change of NMNGs on human chromosome. **(D)** Difference in the expression level of 46 NMNGs between normal and tumor samples. *P<0.05, **P<0.01, ***P<0.001.

The function of 46 NMNGs in STAD is the subject of our upcoming discussion. In the beginning, 46 NMNGs in STAD were examined for CNV and somatic mutations (**Figure 3A**). In 278 out of 433 samples (64.2%), NMNG mutations were found. The TP53 gene had the highest rate of mutation (44%), followed by CDH23 (9%). However, CNV deletions are more likely in EZH2, CDKN2B, and GRP, CNV amplification is more common in IGF1R, KCNB1, and GNAS (**Figure 3B**). The chromosomal positions of NMNG with different copy numbers are shown in **Figure 3C**. In order to ascertain the links between STAD and the expression of NMNG, we also evaluated the mRNA expression levels of 46 NMNGs in both tumor and normal samples. The results showed that 46 NMNGs had different expression patterns. LEPR, AR, PGR, KIT, and VIP were down-regulated in tumor tissues, whereas TP53, GNAS, UCP3, BRCA2, and BRCA1 were up-regulated in tumor tissues (**Figure 3D**).

### Two NMNG patterns of STAD

**Figure 4:**
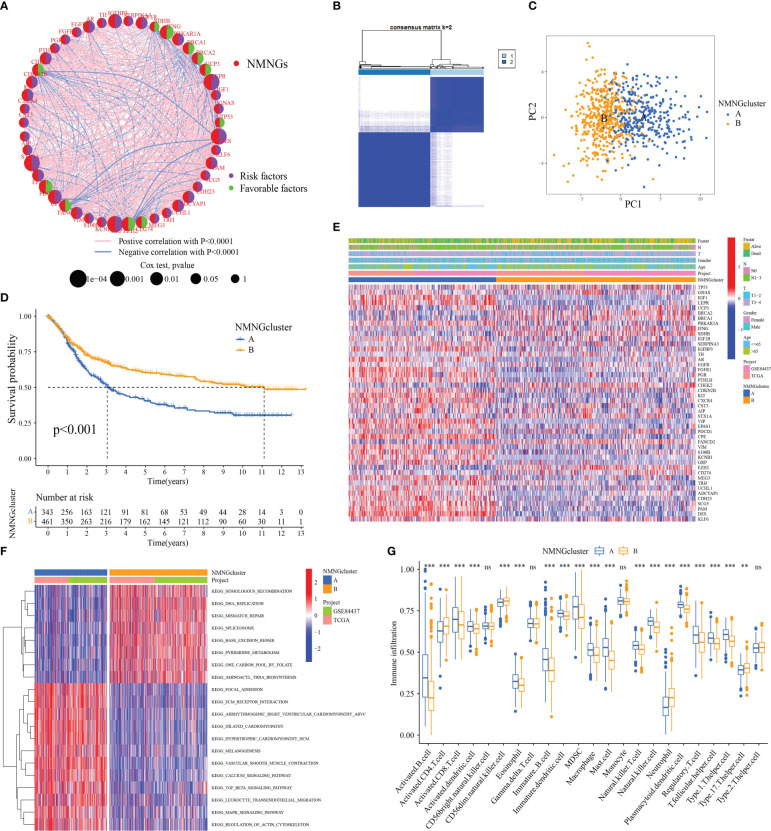
Patterns of NMNG modification and biological features of each pattern. **(A)** Mutual effects of NMNGs in STAD. **(B)** Unsupervised clustering analysis in STAD cohort. **(C)** Principal component analysis for two NMNG modification patterns. **(D)** Survival assay for the two NMNG modification patients. **(E)** Clinical characteristics of two NMNG modification patterns. **(F)** KEGG enrichment assay displaying the stimulation status of biological pathways of two NMNG modification patterns. **(G)** Expression of immune-infiltrating cells in two NMNG modification patterns. ** P<0.01, ***P<0.001; ns, not statistically significant.

The NMNG network outlines the co-expressed relationship among NMNGs with prognostic significances in STAD patients. (**Figure 4A**). The Consensus Cluster Plus R program was utilized to classify patients with various NMNG patterns by quantifying the expression of the 46 NMNGs. Unsupervised clustering was then used to identify two distinct modification patterns (**Figure 4B** and **Supplementary Figure 3**). These two gene clusters were obviously divided (**Figure 4C**). In a subsequent prognostic analysis, it was found that cluster B modification pattern was found to have a better prognosis (**Figure 4D**). **Figure 4E** illustrated the clinical characteristics of the two subgroups of patients. Two NMNG modification patterns were examined using a KEGG enrichment assay to determine their biological roles. It was found that cluster A was significantly enriched in pathways that promote carcinogenesis, including the MAPK signaling pathway, the TGF BETA signaling pathway, the calcium signaling pathway, and the interaction between ECM receptors. DNA replication, mismatch repair, spliceosomes, and base excision repair pathways were enriched in cluster B (**Figure 4F**). We also examined the TME cell invasion of various clusters. According to the analysis, immune cells such as activated B cells, CD8+ T cells, activated dendritic cells, eosinophils, immature B cells, immature dendritic cells, macrophages, mast cells, NK cells, plasmacytoid dendritic cells, regulatory T (Treg) cells, follicular helper T (Tfh) cells, and type 1 helper T (Th1) cells were significantly enriched in cluster A (**Figure 4G**).

### DEGs between distinct NMNG phenotypes

**Figure 5:**
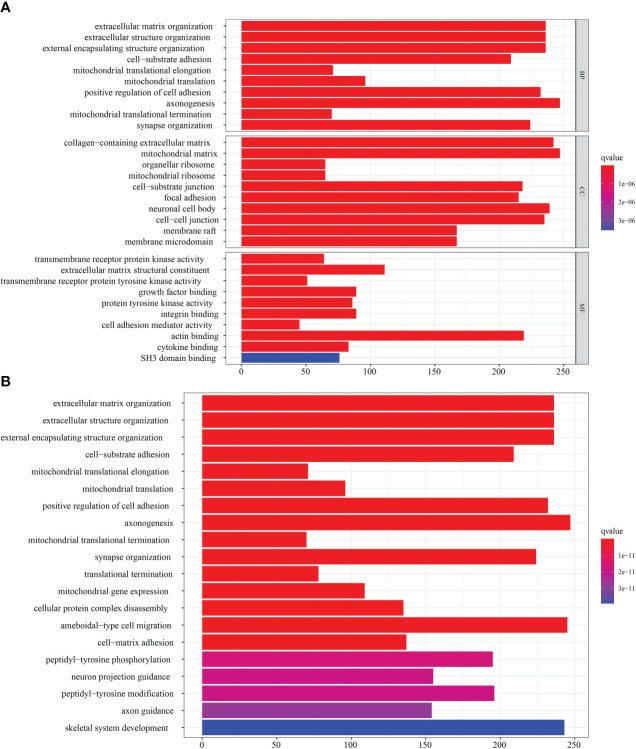
Transcriptome characteristics of NMNG modification patterns in STAD. **(A)** GO enrichment analysis of NMNG DEGs. **(B)** KEGG pathways of NMNG DEGs.

**Figure 6:**
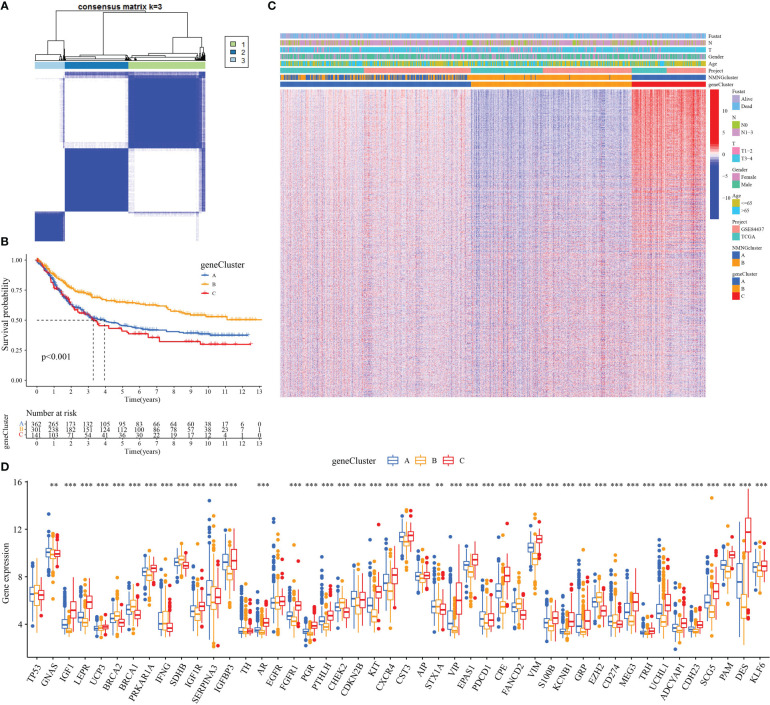
Three genomic subtypes of DEGs with prognostic significance by unsupervised cluster analysis. **(A)** Consensus matrix. **(B)** Survival analysis of gene clusters. **(C)** Different clinicopathological characteristics of these subgroups shown by heatmap. **(D)** Expression of NMNGs in STAD in three gene clusters. **P<0.01 ***P<0.001.

In order to further analyze the molecular mechanisms behind the two NMNG modification patterns, we used the limma package to find 6922 DEGs related to the NMNG phenotype. **Figures 5A, B** display the findings of the analysis of DEG-related enriched GO and KEGG pathways. We used univariate Cox regression analysis on 6922 DEGs to identify 2117 DEGs that affected prognosis for the purpose of further verifying the regulatory mechanism of NMNG modification in STAD. Patients were then divided into three gene clusters based on the results of the unsupervised clustering analysis of these genes (**Figure 6A** and **Supplementary Figure 4**). This implies that there are different NMNG patterns in STAD. The prognosis for cluster C (141 patients) and cluster B (301 patients) patients among the 803 STAD patients was worse (**Figure 6B**). In **Figure 6C**, the various clinicopathological traits of these subgroups are depicted. We found significant variations in NMNG expression across these three clusters (**Figure 6D**).

### Generation of NMNG signatures and functional annotation

**Figure 7:**
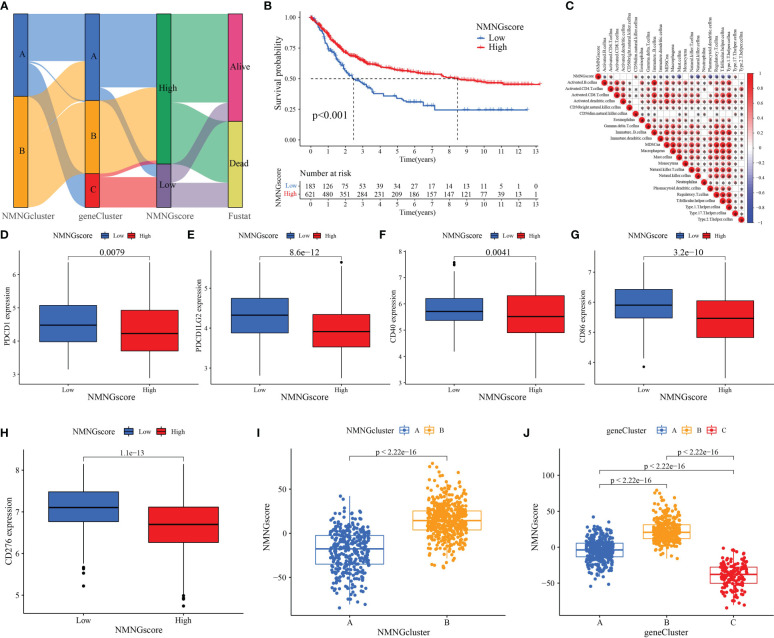
Construction of NMNG signature and exploration of its clinical significance. **(A)** Alluvial diagram showing the changes in NMNGclusters, gene clusters, NMNGscore, and survival status. **(B)** Survival outcomes of patients by NMNGscore. **(C)** Correlation between NMNGscore and immune cells. Blue represents negative correlations and red represents positive correlations. **(D-H)** Expression of immune checkpoints (PDCD1, PDCD1LG2, CD40, CD86, and CD276) among low and high NMNG score groups. **(I)** Variation analysis of NMNGscore between NMNGclusters. **(J)** Variation analysis of NMNGscore between gene clusters. *P<0.05.

These procedures are unable to anticipate the NMNG alteration patterns of specific patients because they only consider STAD patient populations. To assess the pattern of NMNG alterations in each STAD patient, we developed a scoring system, the NMNG score. To see the alterations in several patient parameters, alluvial plots were employed (**Figure 7A**). Then, using the NMNGscore as the parameter, we investigated patient survival results. High NMNGscore patients have greater survival rates (**Figure 7B**). Activated CD4+ T cells, neutrophilia, and Th17 cells were positively correlated with NMNGscore, whereas activated B cells, Eosinophilia, T cells, immature B and dendritic cells, MDSCs, macrophages, mast cells, NKT cells, NK cells, plasmacytoid dendritic cells, Treg cells, Tfh cells, and Th1 cells were negatively correlated (**Figure 7C**). The effect of different ICG expression levels on the TME was subsequently examined. In the group with low NMNGscores, we discovered increased expression levels of ICG, which might have been linked to their worse prognosis (**Figures 7D–H**). Then, we conducted research on the variation in NMNGscore between NMNGclusters and gene clusters. NMNGcluster B and gene cluster B both had significantly higher NMNGscores than NMNFcluster A and gene clusters A and C, accordingly (**Figures 7I, J**). These findings suggested that the NMNGscore can be used to evaluate the pattern of NMNG alteration as well as the tumor’s TME immune cell infiltration features in a particular patient.

### Cancer somatic mutations and patterns of NMNG alteration in TCGA molecular subgroups

**Figure 8:**
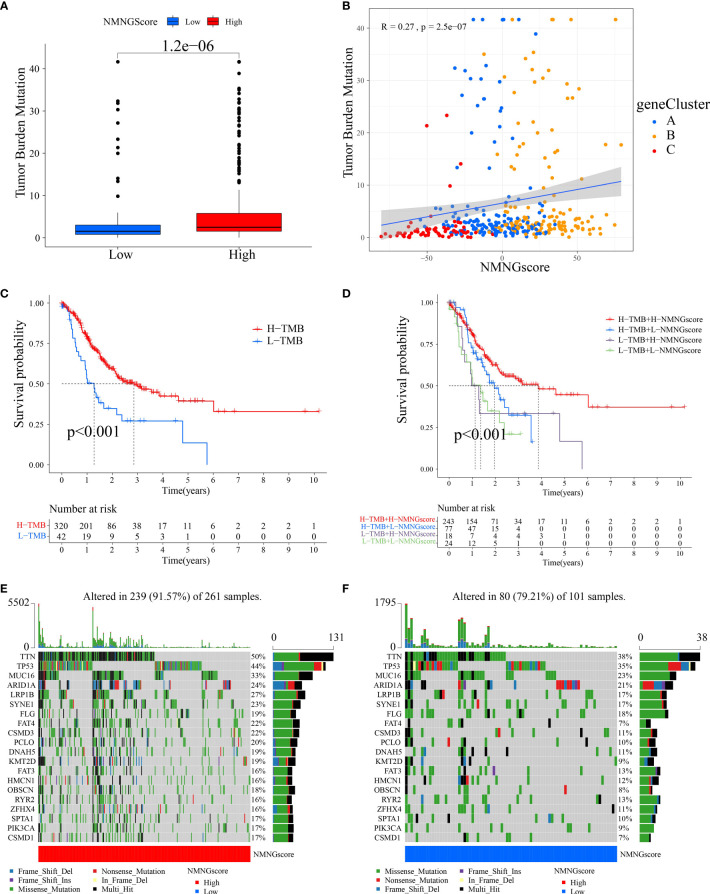
Features of NMNG modification in TCGA molecular subgroups and cancer somatic mutation. **(A)** The distribution differences of the tumor mutational burden (TMB) between low- and high NMNG-score categories in the TCGA-STAD cohort. **(B)** The correlation between NMNG score and TMB. **(C)** Survival assay for low and high TMB groups using KM curves. **(D)** Survival analyses for patients classified by NMNG score and TMB using KM curves. **(E, F)** Waterfall plot of cancer somatic mutations constructed from patients with **(E)** high NMNG score and **(F)** low NMNG score.

Somatic mutations in tumor genomes are related to patients’ response to immunotherapy, according to our studies. As a result, we focused on the distributions of tumor mutation burden (TMB) across distinct NMNGscore categories. The low NMNGscore group had a lower TMB in comparison to the group with a high NMNGscore, and the NMNGscore was positively linked to TMB (**Figures 8A, B**). Furthermore, there was a markedly higher chance of survival in the group of patients with high mutational loads, and this benefit was much more obvious in those patients who also had high NMNGscores (**Figures 8C, D**). The distribution landscape of somatic mutations was then compared between the groups with high and low NMNGscores using the R maftools package. The results showed that the group with a high NMNGscore had a greater TMB than the group with a low NMNGscore (**Figures 8E, F**). Overall, NMNG changes interact with somatic mutations, and NMNGscore categorization may be influenced by chromosomal variance.

### NMNGscore signatures characterized by different immunotherapy landscapes

**Figure 9:**
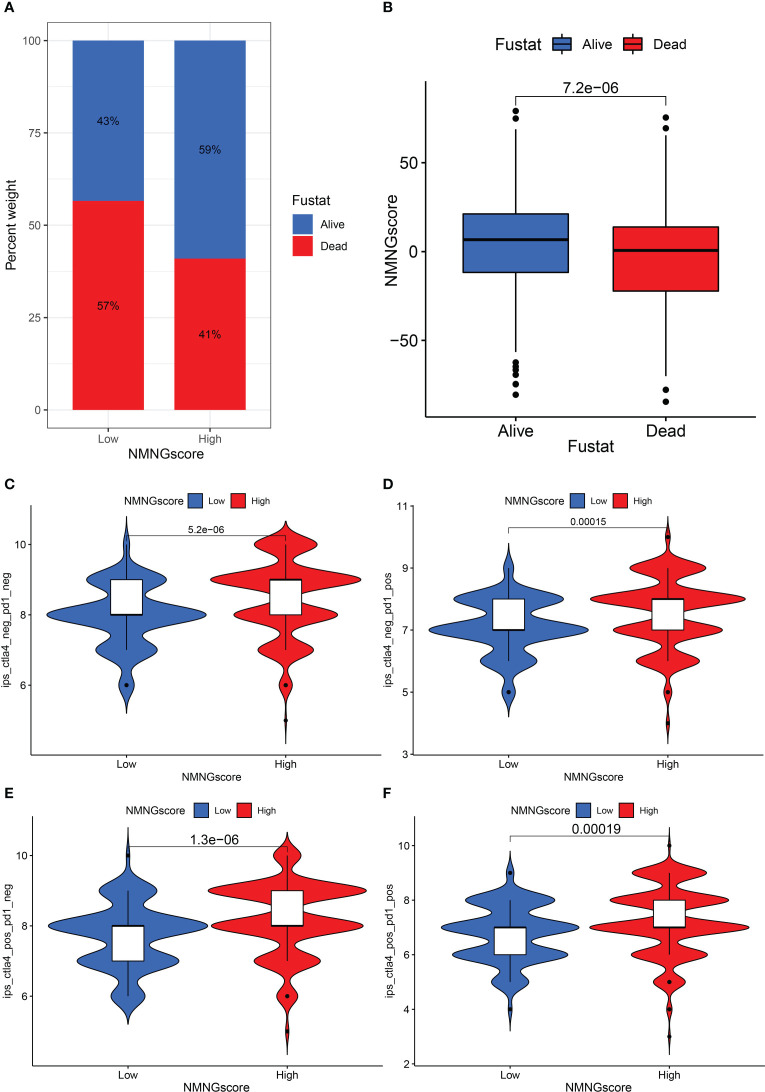
The role of NNMG-scoring signature in immunotherapy. **(A)** The proportion of patients surviving between the low and high NMNG score groups, with a higher proportion surviving in the high scoring group. **(B)** NMNG scores were higher in surviving patients than in patients who died. **(C–F)** The comparison of the relative distribution of immunophenoscore (IPS) between low and high NMNG score groups.

We assessed whether NMNG alterations could predict patient responses to ICIs in the context of the recent approval of drugs that target PD-1 and CTLA-4 for the treatment of various types of cancers. We observed that patients with a high NMNGscore had significant clinical benefits and considerably improved survival status (**Figures 9A, B**). In order to anticipate how efficiently ICIs would work, we also investigated the link between STAD patients’ Immunophenoscore (IPS) and NMNGscore signatures. The variations in treatment outcomes between the groups with high and low NMNGscores were displayed in **Figures 9C–F**. The higher IPS scores in the high NMNGscore group suggest that they are more immunogenic on ICIs. These studies reveal that NMNG scores can be used to estimate how an immunotherapy treatment will affect a patient.

## Discussion

Due to its high morbidity, poor incidence of early diagnosis, and low survival rate, GC poses a severe threat to the health and life of Chinese citizens (28). As of now, early or localized GC should be treated with surgery or endoscopic resection. Nevertheless, 30–40% of GC patients experience recurrence or metastases even after receiving curative resection (29, 30). Several recent studies demonstrate that inhibiting PD-1 is not significantly more effective than chemotherapy (31) and that patients with advanced GC who were administered with PD-1 inhibitors and concurrent chemotherapy have better survival than those who receive chemotherapy alone (32). However, only 15% to 60% of patients receiving anti-PD-1 immunotherapy experience improvement.

During the progression of tumors, abnormal cancer metabolism takes place (33). The metabolism of nucleotides is the last and most important link in the chain of events that contribute to the spread of cancer (5). Recent studies have shown that abnormal nucleotide metabolism suppresses the normal immune response in the tumor microenvironment while accelerating the emergence of tumors (6). Since there are few relevant procedures and studies, the analysis of the relationship between nucleotide metabolism and the development of cancer is growing rapidly. The intervention, alteration, or regulation of molecular pathways linked to aberrant nucleotide metabolism in tumor cells have emerged as novel approaches and concepts for the treatment of malignancies as well as the inhibition of relapse and metastasis (7). The metabolism of tumors is often regulated by the neuroendocrine system, and abnormal neuroendocrine regulation may cause metabolic disorders (19). In addition, numerous studies have demonstrated that nerves’ primary means of modulating tumor cells is through neuroendocrine modulation, which has also been linked to tumor start, development, and a worse prognosis (34–36). Studies show that catecholamine-induced neuroendocrine phenotype of GC cells accelerates GC progression (13). Neuroendocrine regulation also causes corresponding changes in the body’s immune function and thus promotes tumor progression (37). Thus, the NMNG-based prognosis signature of STAD is a promising strategy for prognosis assessment and individual management.

First, we filtered 46 NMNGs to obtain NMNGs that had prognostic significance in STAD. The roles of neuroendocrine and nucleotide metabolism in cancer have been progressively discovered as research into these topics has evolved. As a result, we summarize NMNG variations in a variety of cancers before investigating the role of aberrant nucleotide metabolism and neuroendocrine in STAD. However, partial NMNGs had prognostic values in a variety of cancers and NMNG variations took place more or less often. NMNGs’ genetic abnormalities and variations were also clearly present in a proportion of malignancies. In the majority of tumor types, NMNGs had a positive correlation with TNFA signaling *via* NFKB, KRAS signaling, interferon-gamma response, inflammatory response, and EMT. By controlling the previously stated pathways, abnormal nucleotide metabolism and neuroendocrine may aid in the progression of cancer.

We then focused our research on STAD. In total, 46 NMNGs were enrolled and analyzed in STAD samples. They had an elevated frequency of somatic mutations and CNV change, which had an influence on the NMNG expression. This indicated that STAD-related tumorigenesis and NMNG alteration might be associated. The imbalance of NMNG expression was clearly linked to the onset and progression of STAD due to substantial genetic heterogeneity and expressional alteration landscape between normal and STAD samples in NMNG. Focusing on the interactions between different NMNGs, cross-talk among NMNGs may play a significant role in the development of different NMNG alteration patterns and TME cell-infiltrating aspects in individual tumors.

Based on the expression of 46 NMNGs, STAD samples were divided into two modification pattern clusters with extremely different biological behavior and TME characteristics. NMNG cluster A had a poor prognosis and exhibited significant enrichment in oncogenic activation pathways and TME immune cell infiltration. These findings suggest that NMNG modification patterns have a strong influence on the biological behavior and TME characteristics of individual tumors.

A total of 6922 DEGs were identified between NMNGcluster A and NMNGcluster B by NMNG transcriptional pattern analysis, of which 2117 DEGs of prognostic significance were designated as NMNG-associated signature genes.

Based on 2117 NMNG signature genes we classified STAD samples into three genomic subtypes. Survival analysis showed that gene cluster B had a better survival rate than gene clusters A and C. Therefore, we suggest that NMMNG modifications may help to classify STAD types and to develop appropriate treatment strategies for patients. Then, given the individual heterogeneity of NMNG modifications, we developed a scoring system to assess the pattern of NMNG modifications in individual SATD patients based on NMNG characteristics. We found that patients with high NMNG scores had a higher survival rate.

The difference in the immune state between the high- and low-NMNGscore STAD groups was explored in the following section because the tumor immune milieu may have an impact on tumor therapy. The results showed that NMNGscore was negatively related to activated B cells, Eosinophilna, γδT cells, immature B cells, immature dendritic cells, MDSCs, macrophages, mast cells, NKT cells, NK cells, plasmacytoid dendritic cells, Treg cells, Tfh cells, and Th1 cells. Meanwhile, ICG expression was upregulated in the low NMNGscore group. It is well known that cancer cells are mistakenly considered a normal part of the body and can protect themselves through the immune checkpoint pathway. A worse prognosis was seen in the low-NMNGscore category with a higher proportion of immunological components, indicating the engagement of immune checkpoint mechanisms. The upregulation of PDCD1, PDCD1LG2, CD40, CD86, and CD276 might become promising targets in STAD.

Due to a lack of comprehensive understanding of the immunological milieu in STAD and the inability to determine the immune status of specific individuals, the effectiveness of immunotherapy in STAD has been uneven. It is reported that TMB can be used as an index to predict ICI efficacy and has become a biomarker in certain cancer types to identify patients who will benefit from immunotherapy (38, 39), which may be related to the fact that high TMB may produce more neoantigens recognized by the immune system and trigger a broader anti-tumor immune response (40). We found that the proportion of TMB in the high-NMNGscore subgroup is higher, which also shows that patients in the high-NMNGscore subgroup may benefit more from immunotherapy. In addition, we investigated the relationship between NMNG score and the outcome of CTLA-4/PD-1 inhibitor therapy. We found that the high NMNG score group had a better response to immunotherapy. In conclusion, NMNG modification may be a crucial modulator of the clinical response to immunotherapy, and we indirectly validated the utility of the NMNGscore in predicting immunotherapy responses.

However, we noted some drawbacks related to our research. The construction of our signature was based on retrospective data from the TCGA and GEO. To develop the predictive significance of our prognostic signature, substantial prospective clinical research is needed. Lastly, the signature was developed using bioinformatics research, suggesting that additional basic research is needed to verify our findings.

## Conclusion

In conclusion, this study highlighted the considerable involvement of NMNG modification in characterizing TME infiltration. Our NMNGscore can offer novel insights into clinical decision-making and provide personalized treatment for STAD patients, representing an efficient indicator to anticipate the prognosis of STAD patients.

## Data availability statement

The original contributions presented in the study are included in the article/**Supplementary Material**. Further inquiries can be directed to the corresponding author.

## Author contributions

All authors are solely responsible for the content and writing of the manuscript. YZ and LZ contributed equally to this work. The study’s design, data collection and analysis, article preparation, and manuscript revision all benefited greatly from the efforts of all authors. All authors read and approved the final manuscript.
