# Supplementary figures and images for: Identification and characterization of nucleotide metabolism and neuroendocrine regulation-associated modification patterns in stomach adenocarcinoma with auxiliary prognostic assessment and immunotherapy response prediction
Source: Front Endocrinol (Lausanne). 2023 Jan 16;13:1076521. doi: 10.3389/fendo.2022.1076521 (PMC9885129; doi:10.3389/fendo.2022.1076521)

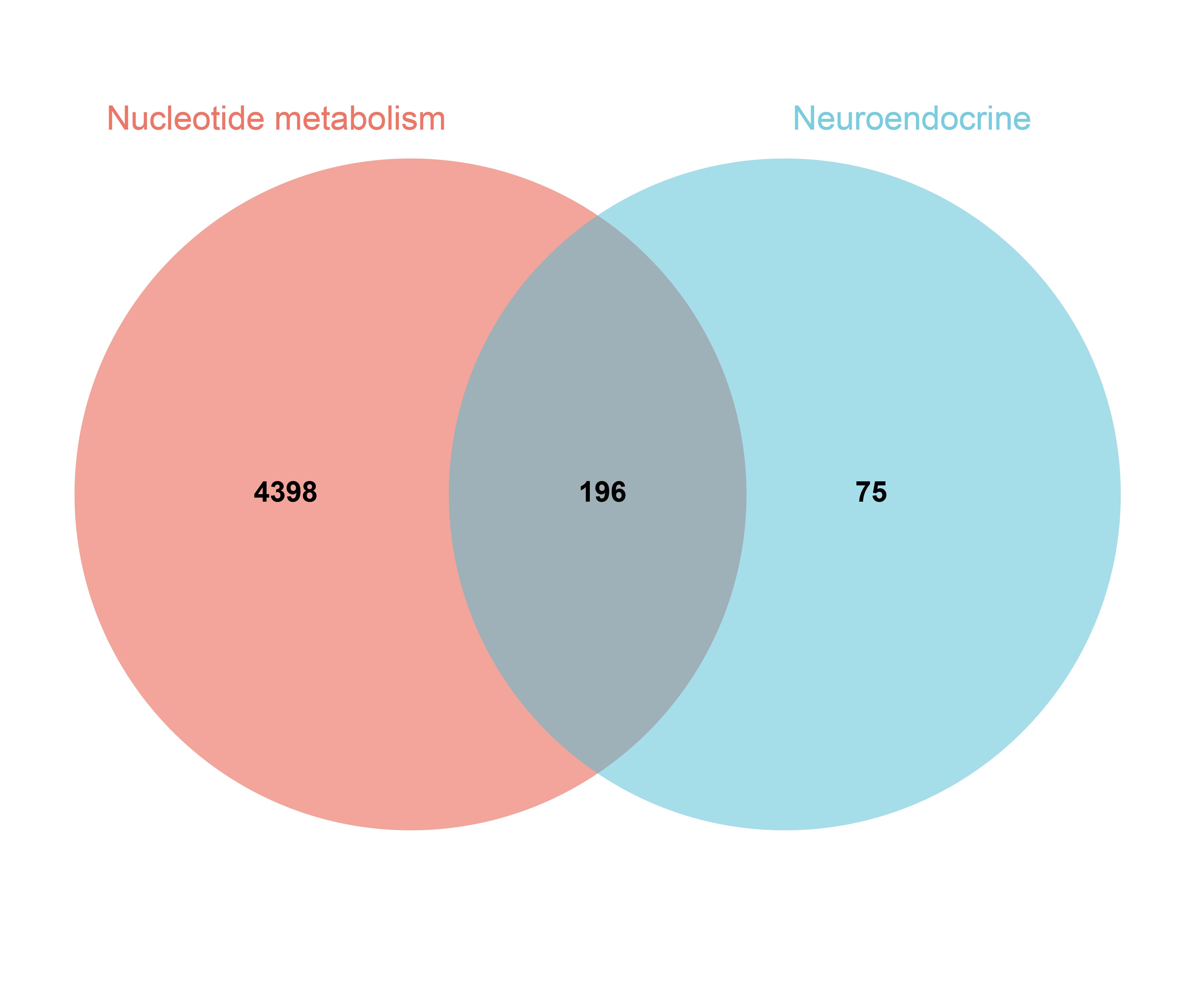

Supplement: Supplementary file 1 [file Image_1.jpeg]

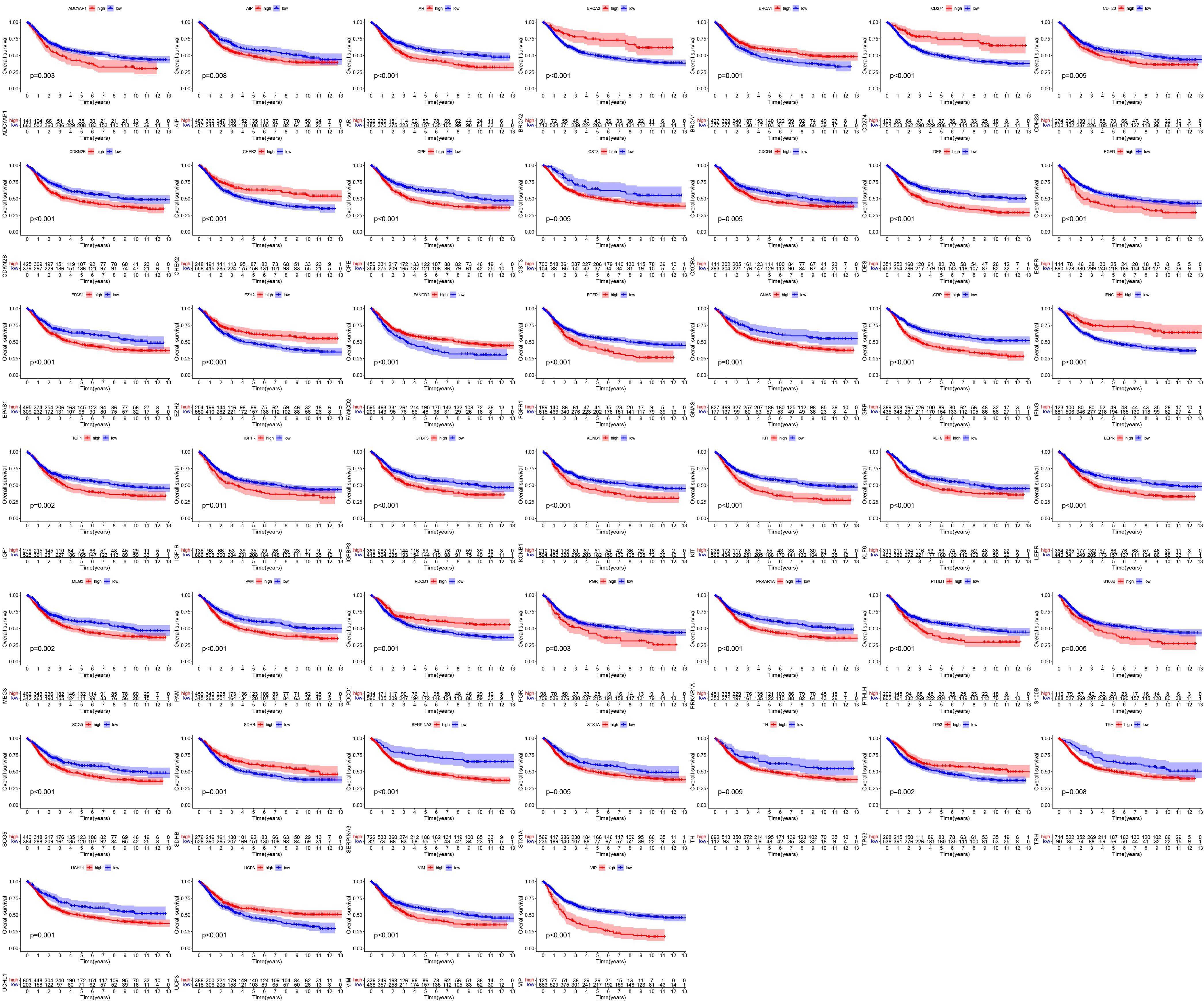

Supplement: Supplementary file 2 [file Image_2.jpeg]

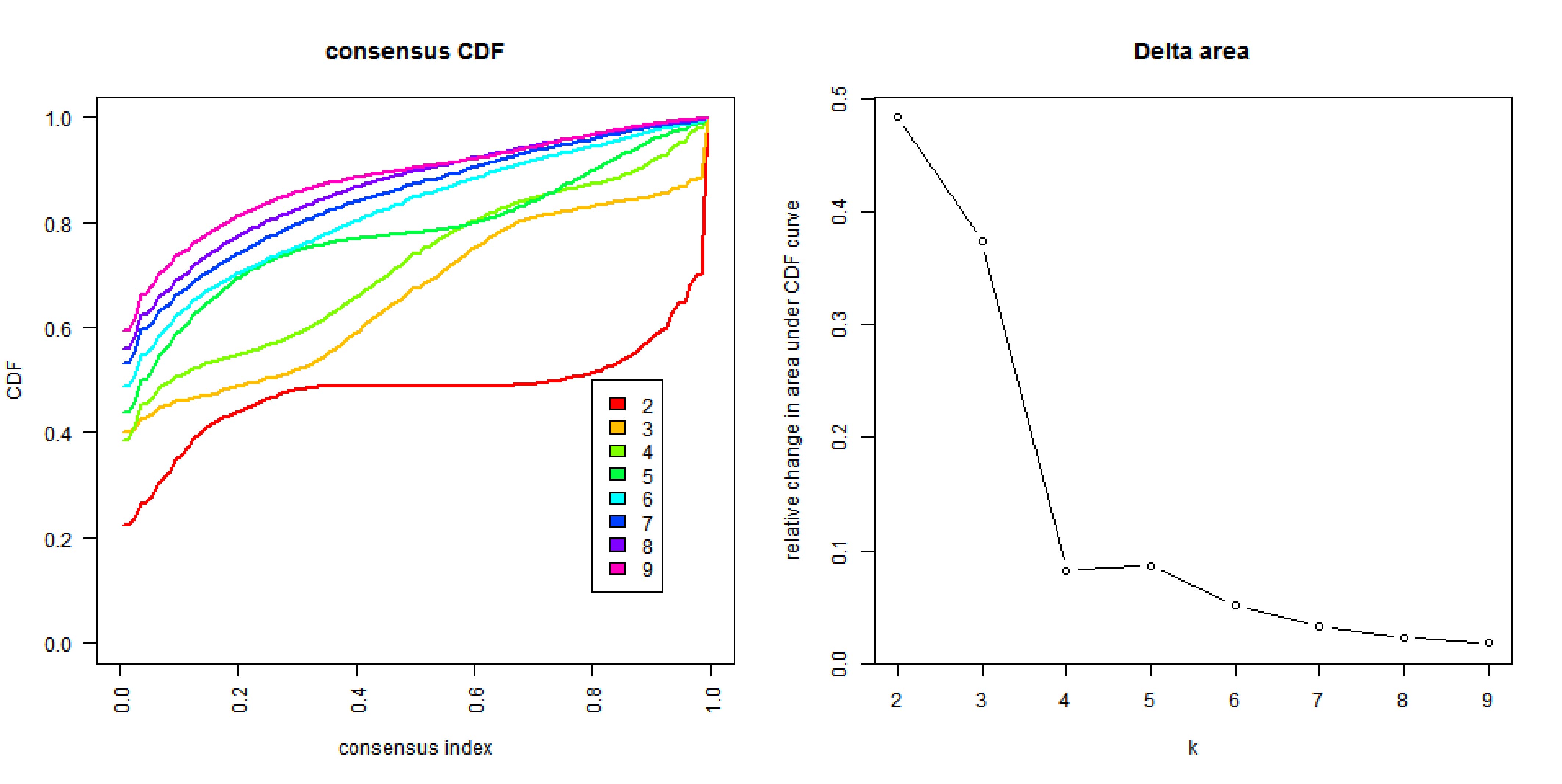

Supplement: Supplementary file 3 [file Image_3.jpeg]

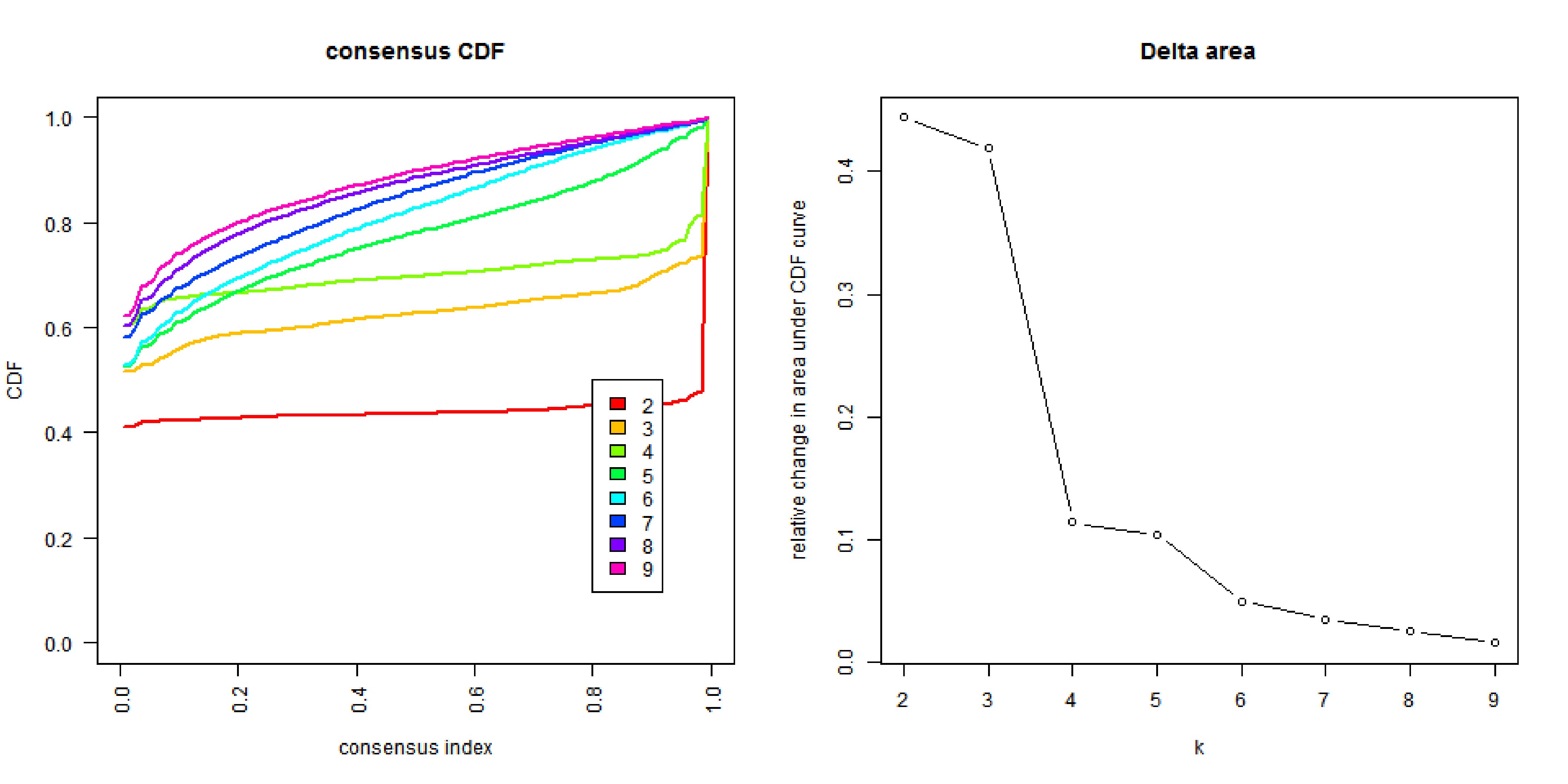

Supplement: Supplementary file 4 [file Image_4.jpeg]
